# Personalized medicine and atrial fibrillation: will it ever happen?

**DOI:** 10.1186/1741-7015-10-155

**Published:** 2012-12-04

**Authors:** Steven A Lubitz, Patrick T Ellinor

**Affiliations:** 1 Cardiovascular Research Center and Cardiac Arrhythmia Service, Massachusetts General Hospital, 149 13th Street, 4th Floor, Charlestown, MA 02129, USA

**Keywords:** Atrial fibrillation, genetics, personalized medicine, risk prediction, pharmacogenetics

## Abstract

Atrial fibrillation (AF) is a common arrhythmia of substantial public health importance. Recent evidence demonstrates a heritable component underlying AF, and genetic discoveries have identified common variants associated with the arrhythmia. Ultimately one hopes that the consideration of genetic variation in clinical practice may enhance care and improve health outcomes. In this review we explore areas of potential clinical utility in AF management including those relating to pharmacogenetics and risk prediction.

## Introduction

Atrial fibrillation (AF) is a common cardiac arrhythmia marked by loss of coordinated atrial electrical and mechanical function. Among the potential consequences of AF are symptoms and decreased functional status [1], as well as increased risks of thromboembolic stroke, heart failure, and mortality [2]. Thus far, efforts to prevent AF have generally been limited, and therefore management is directed at preventing or alleviating adverse consequences of the arrhythmia.

In recent years, heritability underlying AF has been recognized [3-7]. AF risk is increased by about 40% if a first-degree relative has AF, and two-fold if that relative develops AF by age 65 [7]. Numerous genetic variants associated with AF have been reported through traditional mapping techniques such as linkage and candidate gene analyses [8]. For example, novel gain-of-function mutations in *KCNE1 *were recently described in this journal using a candidate gene approach in patients with early onset AF [9]. *KCNE1 *encodes the beta subunit of the potassium channel comprising the I_Ks _current, which governs atrial repolarization. Such gain-of-function mutations, and their expected shortening of the action potential duration, are consistent with electrical reentry as a predominant pathophysiological mechanism underlying AF [10]. Mutations in sodium, potassium, and calcium channel subunits, as well as in gap junction and non-ion channel proteins have been reported and are reviewed elsewhere (Table 1) [8,11].

**Table 1:** Atrial fibrillation (AF) susceptibility genes and loci

AF susceptibility genes			AF susceptibility loci		
**Gene**	**Mechanism**	**Ref**.	**Chromosomal locus**	**Candidate gene**	**Ref**.

Potassium channel			Large-scale candidate gene or genome-wide association study		
*KCNQ1*	Enhanced repolarization (increased I_Ks_)	[61-65]	1q21	*IL6R*	[66]
*KCNE1*	Enhanced repolarization (increased I_Ks_)	[9,67-69]	1q21	*KCNN3*	[15,16]
*KCNE2*	Enhanced repolarization (increased I_Ks_)	[70]	1q24	*PRRX1*	[16]
*KCNE5*	Enhanced repolarization (increased I_Ks_)	[71]	4q25	*PITX2*	[12-16]
*KCNH2*	Enhanced repolarization (increased I_Kr_)	[72,73]	7q31	*CAV1*	[16]
*KCNJ2*	Enhanced repolarization (increased I_K1_)	[74]	9q22	*C9orf3*	[16]
*KCNA5*	Delayed repolarization and afterdepolarizations (decreased I_Kur_)	[75]	10q22	*SYNPO2L/MYOZ1*	[16]
Sodium channel			14q23	*SYNE2*	[16]
*SCN5A*	Hyperpolarizing shift in inactivation (loss-of-function)/depolarizing shift in inactivation (gain-of-function)	[76-81]	15q24	*HCN4*	[16]
*SCN1B*	Decreased I_Na _current and altered channel gating	[82]	16q22	*ZFHX3*	[16]
*SCN2B *	Decreased I_Na _current and altered channel gating	[82]	Familial loci without known gene		
Ion channel-related			6q14-q16	*-*	[83]
*GJA5*	Impaired cellular transport and intercellular electrical coupling, increased dispersion of refractoriness	[84-87]	10p11-q21	-	[88]
*ANK2*	Loss-of-function reduces expression and membrane targeting of Ca_v_1.3 (decreased I_Ca,L_)	[89,90]	10q22-q24	-	[91]
Non-ion channel					
*LMNA*	Disruption of nuclear function or altered interaction with cytoplasmic proteins	[92-95]			
*NUP155 *	Reduced nuclear membrane permeability, enhanced repolarization	[96,97]			
*AGT*	Unknown	[98,99]			
*ACE*	Insertion/deletion, unknown mechanism	[100-102]			
*NPPA*	Mutant peptide, enhanced repolarization	[103]			

AF susceptibility genes were based on prior linkage mapping or candidate gene sequencing efforts. AF susceptibility loci were based on candidate gene chip or genome-wide association studies.

Large-scale genome-wide association studies have identified nine common AF susceptibility loci (Table 1) [12-16]. In aggregate, these discoveries implicate cardiac ion channels, transcription factors central to cardiopulmonary development, and signaling molecules as common pathways involved in the development of AF. Prior to these genetic discoveries, the relations between many of these pathways and AF pathogenesis were unrecognized.

Genetic associations with AF have spawned an exciting new era of biological investigation. Integrating recent discoveries with previous insights about atrial remodeling and AF pathophysiology has led to resurgence of interest in exploiting biological pathways with pharmaceuticals for AF management [17,18]. Given the strong heritable component underlying AF, these discoveries also have elicited hope that determining genetic variation in patients will help individualize clinical management and improve health outcomes. The concept of applying knowledge of an individual's genotype to clinical management is commonly referred to as personalized or precision medicine.

In many ways, as with most areas of clinical medicine, the management of patients with AF is already personalized [19,20]. Prescription of thromboembolism prophylaxis regimens is tailored for each patient. Bedside stroke and bleeding risk prediction rules [21-24] are available to help clinicians decide whether to prescribe aspirin or systemic anticoagulants. Cardioversions, antiarrhythmic therapy, and ablation for AF are variably prescribed depending on patient symptoms. When choosing which systemic anticoagulants or antiarrhythmic medications to prescribe, clinicians consider comorbidities, drug efficacy, adverse effects, contraindications, pharmacologic interactions, and cost. Ablation procedures for patients with persistent AF are more extensive than for those with paroxysmal AF [25].

Despite the personalized nature of AF management, care of patients with AF is challenging owing to the complexity of the arrhythmia. Patient management is limited by unpredictable recurrence and progression of AF, differing susceptibilities to stroke and heart failure, and variable treatment responses. These challenges are opportunities for diagnostic and therapeutic innovation, and represent areas where genetic medicine might facilitate clinical management. Two promising areas for the near-term application of genetic information in AF include pharmacological management and risk prediction (Table 2).

**Table 2:** Potential areas for clinical application of genetic discoveries in atrial fibrillation (AF) management

Area	Example	Potential utility	Limitations
Risk prediction	Predict new-onset AF or AF-related morbidity (stroke, heart failure, mortality risk)	Benchmark for clinical trial development	Absence of known preventive strategies for AF
		Facilitate AF and clinical outcome prevention	Cohorts for genetic risk score derivation predominantly of European ancestry
			Small relative risks of discovered variants
			Complexity of incorporation into clinical practice
			Cost
			Untested
	Predict AF progression	Early antiarrhythmic or ablation intervention	Unclear relations between AF progression and morbidity and mortality
			Untested
Patient management	Pharmacogenetics	Maximize efficacy, minimize adverse effects	Development of novel agents with wide therapeutic margins
		Thromboembolism prophylaxis	Ablation and mechanical interventions as alternatives
		Antiarrhythmic therapy	Complexity of incorporation into clinical practice
			Cost
			Untested

## Potential areas for clinical application of genetic information

### Pharmacogenetics

Many medications used for AF treatment have narrow therapeutic margins. Antiarrhythmic efficacy is only about 50% at 12 months [26], and prohibitive adverse effects such as proarrhythmia or other reactions may occur [27,28]. Thromboembolism prophylaxis with warfarin is limited by increased risks of disabling stroke among subtherapeutic patients, and bleeding among those with supratherapeutic doses [29].

### Can the effectiveness and safety of pharmacologic agents used in AF be improved by utilizing genetic information?

Pharmacogenetics refers to how genetic variation accounts for differences in drug responses among individuals. Measurable differences in pharmacodynamics (that is, the interaction of drugs with their target molecules) and pharmacokinetics (that is, properties relating to drug uptake, distribution, duration of effect, metabolism, excretion, and so on) are likely to be associated with genetic variation. In a recent proof-of-concept paper, whole genome sequencing in a single individual identified over 60 previously reported pharmacogenetic variants affecting drug response [30].

A high-profile example of a pharmacogenetic effect relevant to AF management relates to warfarin. Warfarin dose requirements have been linked to variation in *CYP2C9 *[31] and *VKORC1 *[32], genes encoding products involved in warfarin pharmacokinetics and pharmacodynamics, respectively. Dosing algorithms that include genetic variation in these genes explain a striking one-third to one-half of the variability in warfarin dose requirements [33]. Accounting for variants in these genes may be particularly beneficial (1) during initiation of warfarin therapy when complications relating to excessive or underdosing are particularly prevalent, and (2) for identifying individuals with unexpectedly high or low therapeutic dose requirements [34] who may therefore be at elevated risk for thromboembolism or bleeding, respectively. The US Food and Drug Administration has advised physicians that genetic testing may improve initial dosing estimates among patients taking warfarin [35] and dosing prediction algorithms are available.

Nevertheless, routine warfarin testing has not been widely adopted for several reasons. Novel anticoagulants [36] and procedures [37] are emerging as potential alternatives to warfarin, rigorous surveillance may improve time spent in the therapeutic range thereby improving safety and efficacy [38], and logistical considerations such as costs and genetic literacy pose challenges to the adoption of genetic testing. Currently, the safety and efficacy of genotype-guided warfarin dosing is being compared to routine dosing in the ongoing Clarification of Optimal Warfarin Dosing through Genetics (COAG) trial (clinicaltrials.gov identifier NCT00839657).

Regardless of the outcome of this trial, the striking associations between genetic variants and warfarin doses have revealed the clinical potential for pharmacogenetics. Emerging reports demonstrate other associations between genetic variants and drug response. Genetic variation has been reported to associate with response to β-adrenergic antagonism in patients with AF [39], and genetic variation associates with drug-induced torsade de pointes [40,41]. Given the heterogeneity in the molecular mechanisms underlying AF [42], as well as variability in medication efficacy and adverse effects, there may be a practical role for pharmacogenetics in the management of patients with this arrhythmia. It is worth noting that genetic variation has also been reported to associate with AF recurrence after catheter ablation [43]; however, the role of genetic testing for a procedure in which success is dependent on many technical factors remains undefined.

The next steps for assessing the clinical utility of pharmacogenetics will involve performing systematic phenotyping, genotyping, and association testing of established and novel pharmacological agents used for AF management. Candidate genes that ought to be prioritized for screening include those encoding products involved in drug uptake or metabolism, and those encoding drug targets.

### Risk prediction

Risk prediction encompasses estimation of AF probability and prediction of AF-related complications such as stroke, heart failure, and death. The goal of prediction algorithms is to identify individuals prior to the onset of AF or AF-related morbidity, and thereby help target prevention efforts. Estimation of risk may also assist with clinical trial development by enabling enrollment of individuals in a particular risk stratum.

Clinical prediction algorithms for new-onset AF have been developed in community-based cohorts of European and African American ancestry [44-46]. These algorithms have good but not excellent discriminative properties. In a given sample of individuals without AF, they correctly assigned a higher predicted risk to those who ultimately developed AF only about 60% to 70% of the time [44-46]. Simply flipping a coin would correctly assign a higher risk to those developing AF 50% of the time.

### Can genetic discoveries improve risk prediction?

We and other investigators from the Framingham Heart Study tested whether accounting for the occurrence of AF in a first-degree relative (familial AF) improved AF risk prediction beyond clinical risk factors [7]. AF prediction improved slightly after accounting for familial AF. In a separate study from Sweden, adding genotypes for two common polymorphisms at the chromosome 4q25 and 16q22 AF susceptibility loci to clinical risk factors did not significantly improve AF risk prediction [47].

For most complex conditions, genetic risk scores have not achieved substantial improvements in discrimination over scores comprised of clinical risk factors. Yet enhancements in the understanding of genetic risk modeling reveal insights that may improve the utility of genetic risk scores in the future. For example, variants included in genetic risk scores to date have small effect sizes (for example, relative risks of 1.1 to 1.5). Single risk factors with such modest relative risks are unlikely to have meaningful impacts on discrimination alone [48]. Large-effect variants, which have greater potential to improve discrimination, are rare and not captured by genotyping panels used in genome-wide association studies.

Fine mapping and sequencing offer promise for the discovery of such large-effect variants [49]. For example in a fine mapping analysis we previously identified independent genetic variants at the chromosome 4q25 locus associated with AF [50]. A multimarker risk score comprised of genotypes tagging these three independent signals demonstrated that AF risk increased with an increasing number of AF risk alleles. The multimarker score identified about 12% of individuals with a twofold or greater relative risk for AF, and 1% with an estimated sixfold elevated risk of AF compared to those with the most common genotypes. This is one of the largest relative risks to date for a qualitative trait in the era of genome-wide association studies.

Another potential reason that genetic risk scores have not substantially improved prediction models is that few variants were tested. Accounting for additional genetic variants associated with conditions at far less stringent significance thresholds can substantially increase the proportion of variance explained by genetic factors [51-53]. In simulations, genetic profiling using large numbers of genetic variants significantly improved risk discrimination [54].

Novel metrics have been developed to assess clinical utility of prediction models, with increased recognition that measures of discrimination alone can be insensitive [55]. The role of novel prediction metrics with respect to genetic profiling has not been extensively explored.

Furthermore, although a widespread heritable component underlies AF, the arrhythmia may result from a variety of different pathological processes. Whether genetic profiling will successfully predict incident AF may depend on the extent to which the 'type' of AF studied is influenced by genetic factors. The importance of refining AF classification has been highlighted elsewhere [56,57], and may overcome the potential challenges that phenotypic heterogeneity presents to risk prediction efforts.

The absence of data demonstrating clinical utility of routinely using genotype information to guide clinical management in AF should not deter hope at this stage, but rather should be viewed as an opportunity for systematic testing of the clinical role of genetic information. Nevertheless, there is a risk that hype can obscure the monumental insights gained from the current era of genetic discovery. As an example, companies such as deCODE and 23andMe are performing direct to consumer genetic testing, and purport to calculate one's genetic risk of AF based on one or a few polymorphisms associated with modestly increased relative risks of AF. These calculations are not based on well-defined genetic effect estimates, and do not consider other genetic risk factors, interactions between genes, the predictive utility of genetic risk markers, or competing risks. Moreover, in the absence of data regarding clinical risk factors and environmental exposures, how are these results to be interpreted? Problems with direct to consumer genetic testing are reinforced by recent literature highlighting the facts that patients often have misperceptions about the results genetic testing [58], and practitioners frequently lack confidence in explaining direct to consumer genetic testing results [59].

## Summary

Owing to the absence of currently available outcome data, there is insufficient evidence to recommend routine testing for genetic variation in the management of patients with AF, as was highlighted by a recent Heart Rhythm Society and European Heart Rhythm Association consensus statement on genetic testing [60]. However, increased attention to pharmacogenetics and prediction modeling using genetic information offers hope for improved clinical care. The absence of data demonstrating direct utility of incorporating genotype information into clinical practice provides an opportunity to systematically assess whether applying genetic data to clinical practice will enhance outcomes.

As with any new technology or development, systematic assessment of its utility in clinical medicine is warranted. Such assessment includes identifying suboptimal realms of clinical care, determining whether such areas present opportunities for application of genetic knowledge, testing whether genetic discoveries are superior to standard care for improving health outcomes, and evaluating whether the expected benefits of implementing genetic testing justify the resource utilization that would be necessary for its adoption.

Recent AF genetic discoveries herald a sea change in the approach to AF research. Investigation of the pathogenic mechanisms underlying this common and morbid arrhythmia is now informed by unbiased techniques, allowing investigators to branch out from preconceived biological mechanisms and reliance on established animal models of AF. It will take many years to realize the full impact of recent genetic discoveries. In the meantime, questions surrounding the utility of applying genetic discoveries to patient management will only be clarified by rigorous testing of genetic information in clinical practice. Ultimately, it is the opportunity for innovation, discovery, and improvement in health outcomes that makes this era of genetic discovery so exciting.

## Competing interests

The authors declare that they have no competing interests.

## Authors' contributions

SAL and PTE were involved in drafting the manuscript and revising it critically for important intellectual content. Both gave final approval of the manuscript.

## Authors' information

Steven Lubitz, MD, MPH is a cardiac electrophysiologist at the Massachusetts General Hospital and Instructor in Medicine at Harvard Medical School. Patrick Ellinor, MD, PhD is a cardiac electrophysiologist at Massachusetts General Hospital and Associate Professor at Harvard Medical School.

## Pre-publication history

The pre-publication history for this paper can be accessed here:

http://www.biomedcentral.com/1741-7015/10/155/prepub
